# Supplementary material for: Personalized visual encoding model construction with small data
Source: Commun Biol. 2022 Dec 17;5:1382. doi: 10.1038/s42003-022-04347-z (PMC9759560; doi:10.1038/s42003-022-04347-z)
Supplement: Supplementary file 2 — Supplementary Information [file 42003_2022_4347_MOESM2_ESM.pdf]

Supplementary Information for manuscript: *Personalized visual encoding model construction with small data*

Zijin Gu,[1] Keith Wakefield Jamison,[2] Mert R. Sabuncu,[1] Amy Kuceyeski [2]*

[1]School of Electrical and Computer Engineering, Cornell University, Ithaca, New York, USA
[2]Department of Radiology, Weill Cornell Medicine, New York, New York, USA
*To whom correspondence should be addressed; E-mail: amk2012@med.cornell.edu.

Supplementary Figures

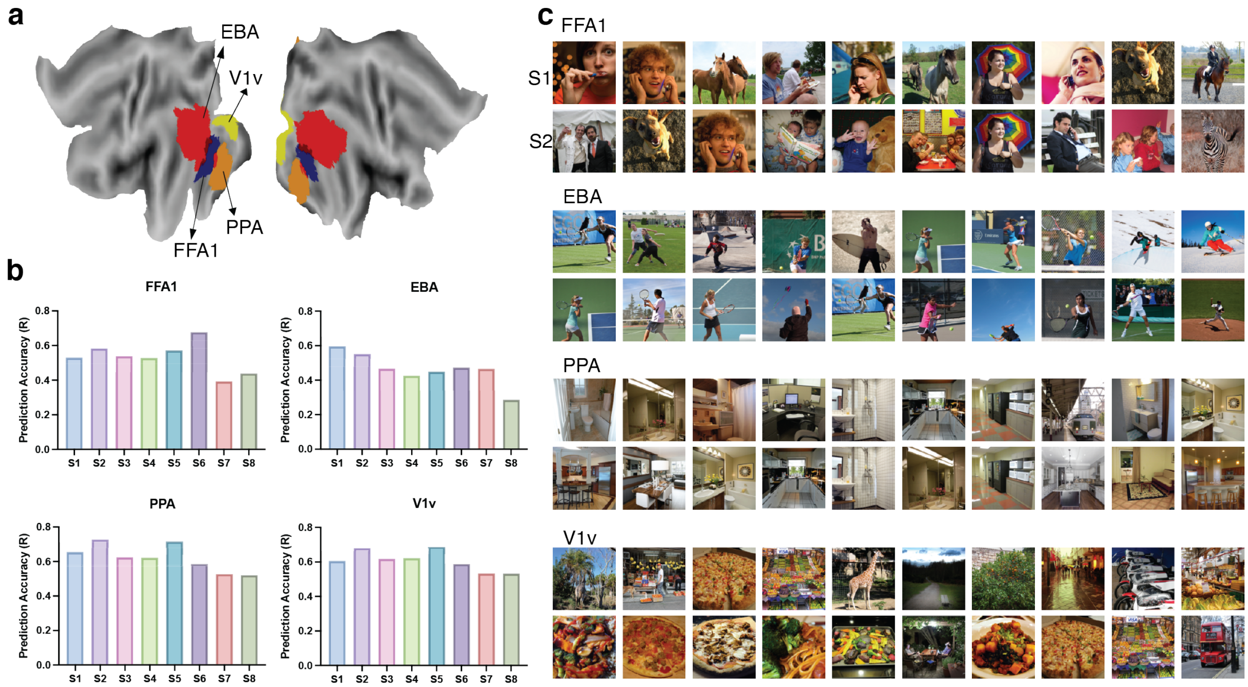

Supplementary Figure 1: Prediction performance of individual-20K models. **a** The four regions for which we built encoding models - one early visual region (V1v) and three higher order regions (FFA1, EBA and PPA). **b** Individual-20K accuracies of each of the 8 NSD subjects (indicated by x-axis labels S1 to S8) for FFA1, EBA, PPA and V1v. **c** Top 10 images that maximize the predicted activation in FFA1, EBA, PPA and V1v for S1 and S2.

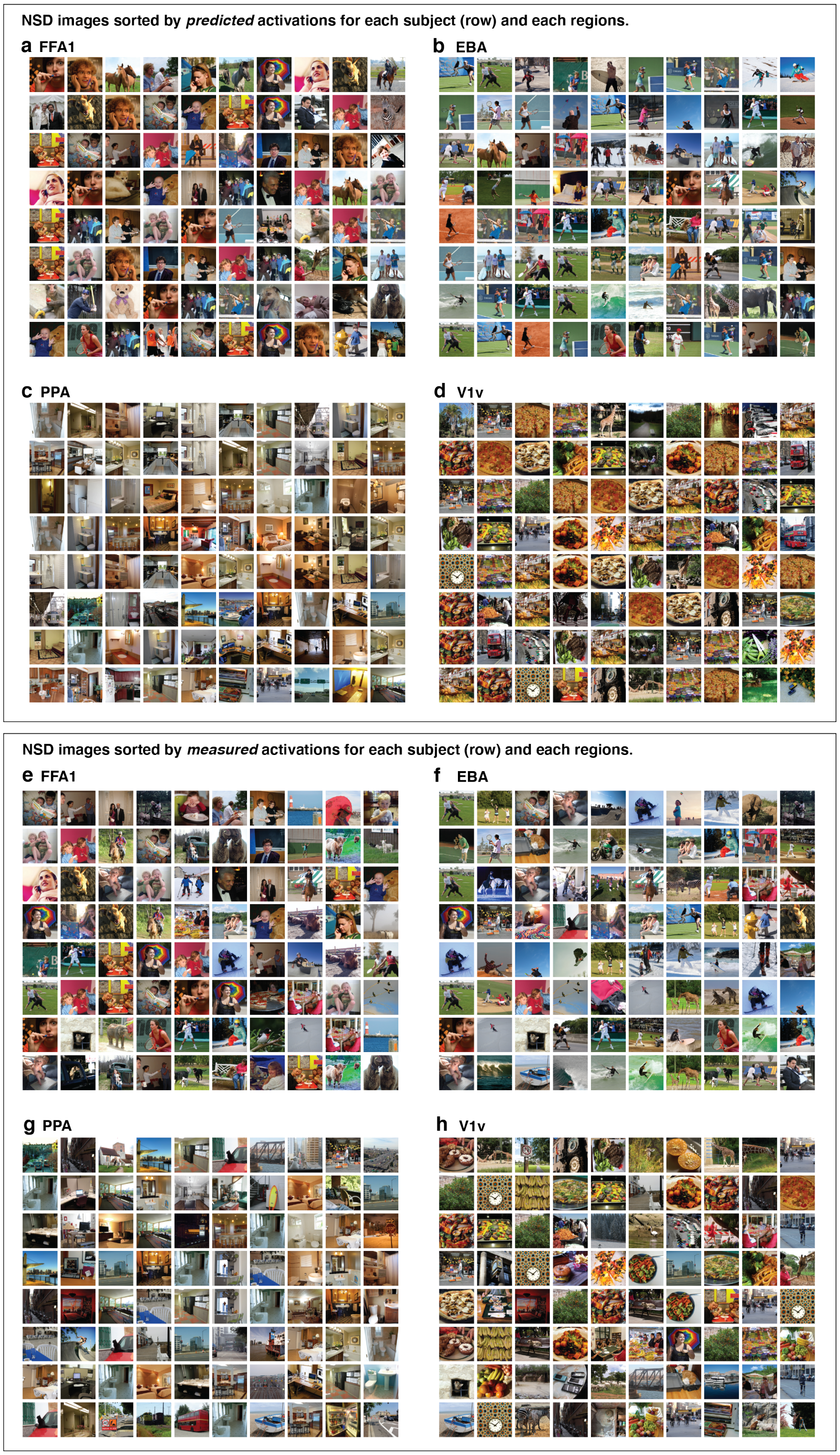

Supplementary Figure 2: Top 10 images for NSD subjects. **a**, **b**, **c**, **d** Images that give highest predicted activation in FFA1, EBA, PPA and V1v regions using the individual-20K encoding model. **e**, **f**, **g**, **h** Images that give highest predicted activation in FFA1, EBA, PPA and V1v regions using the fMRI measurement.

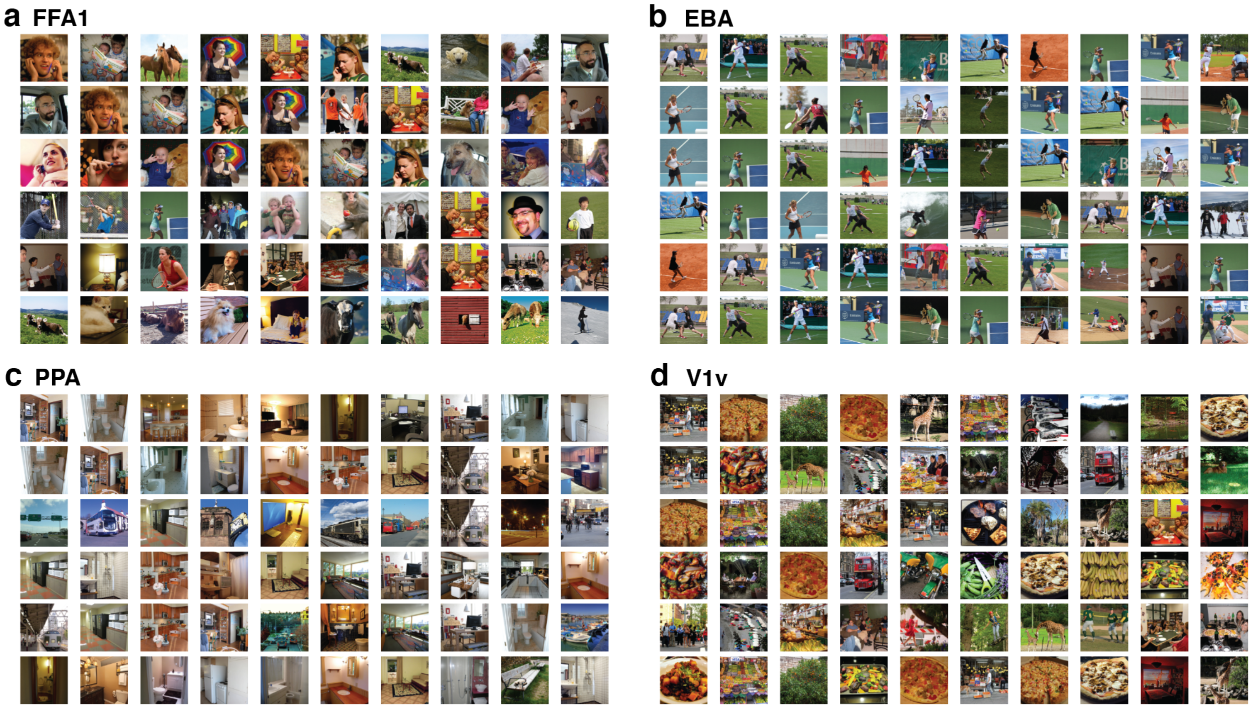

Supplementary Figure 3: Top 10 images for 6 NeuroGen subjects. **a**, **b**, **c**, **d** Images that give highest predicted activation in FFA1, EBA, PPA and V1v regions using the linear ensemble encoding model.

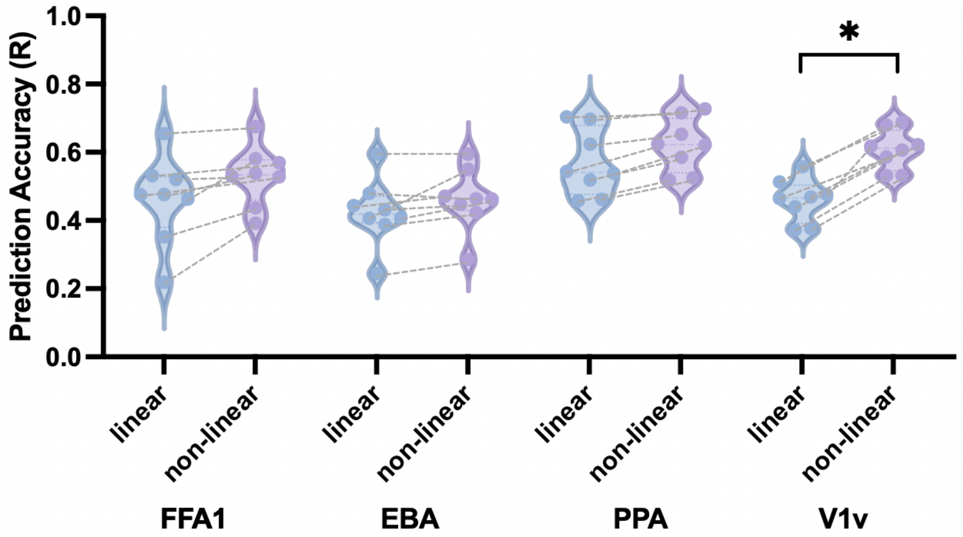

Supplementary Figure 4: Comparison of linear encoding model (ImageNet feature extractor with fixed, pre-trained weights) and non-linear encoding model (ImageNet feature extractor weights were finetuned on the individual's data).

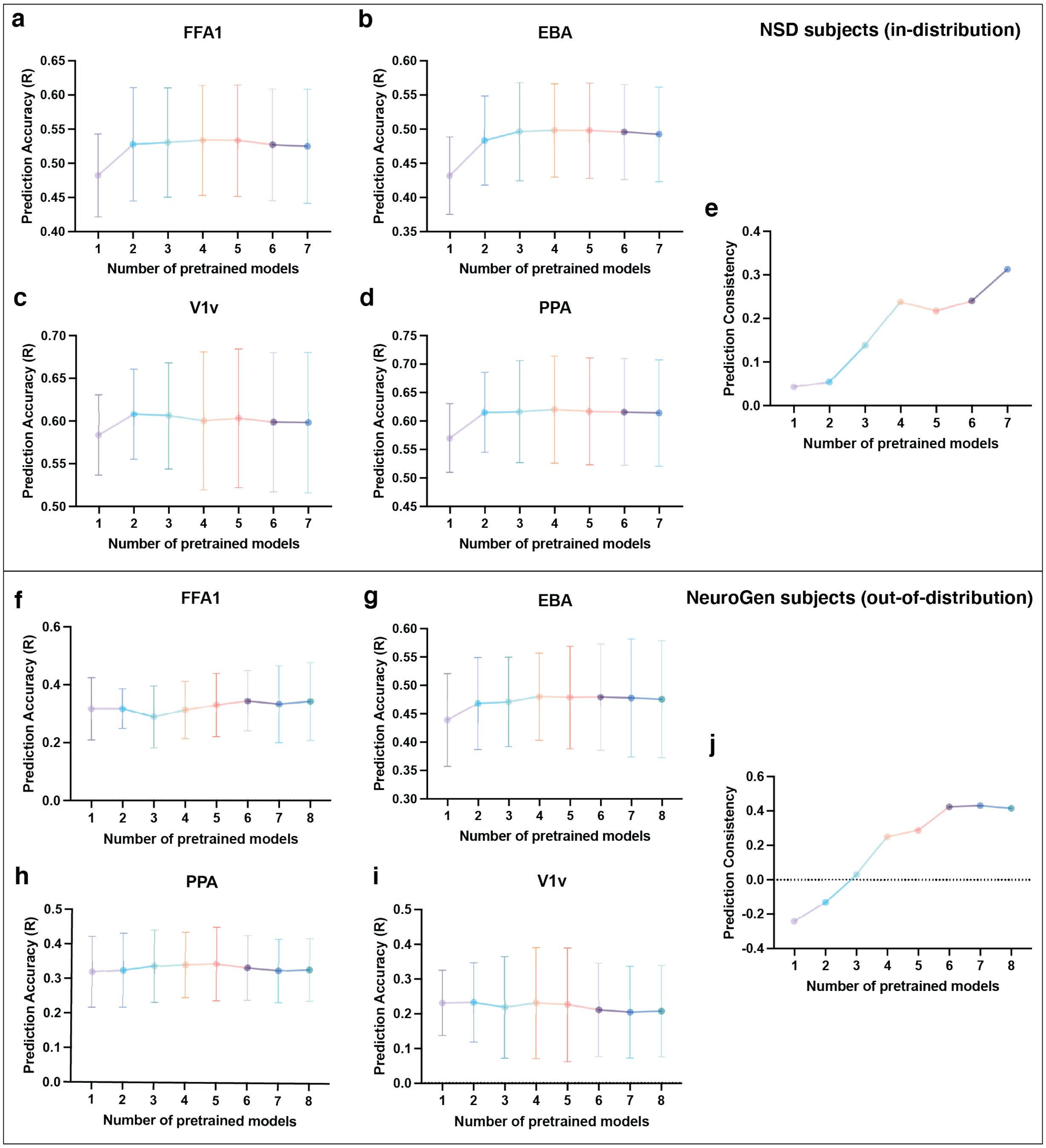

Supplementary Figure 5: Relationship between number of pretrained encoding models included in the linear ensemble approach and prediction accuracy and prediction consistency. **a,b,c,d,e** NSD subjects. **f,g,h,i,j** NeuroGen subjects. Error bars represent the standard deviation of the group of subjects.

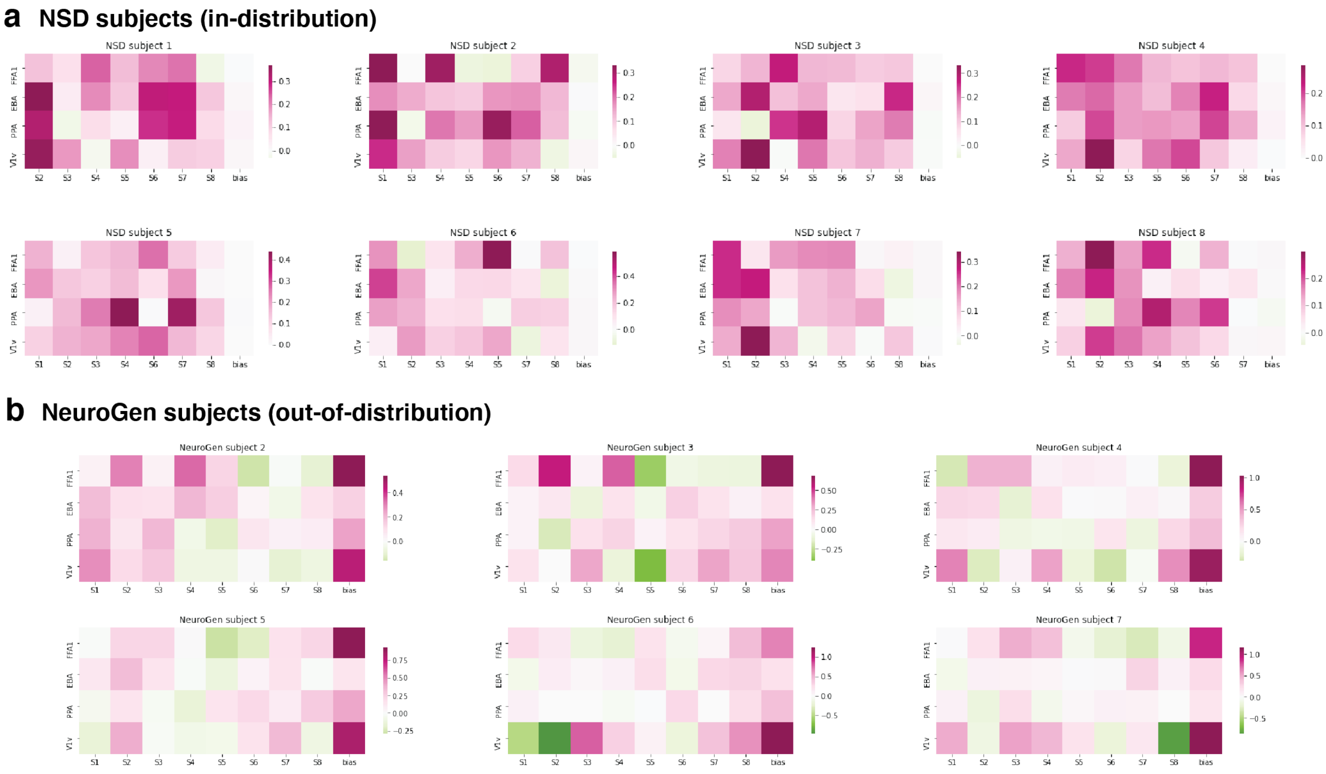

Supplementary Figure 6: Weights for the linear ensemble models. **a**NSD subjects; **b** NeuroGen subjects.

a OFA top 25 images

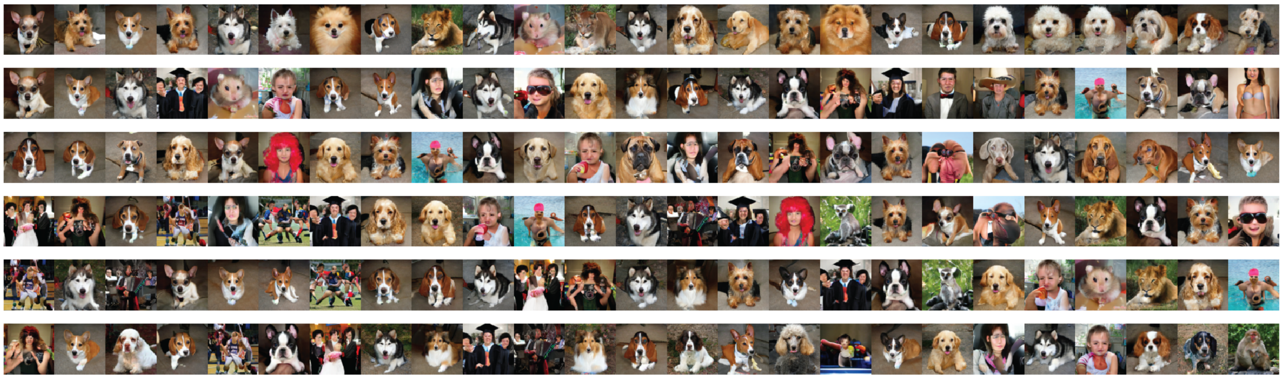

b FFA1 top 25 images

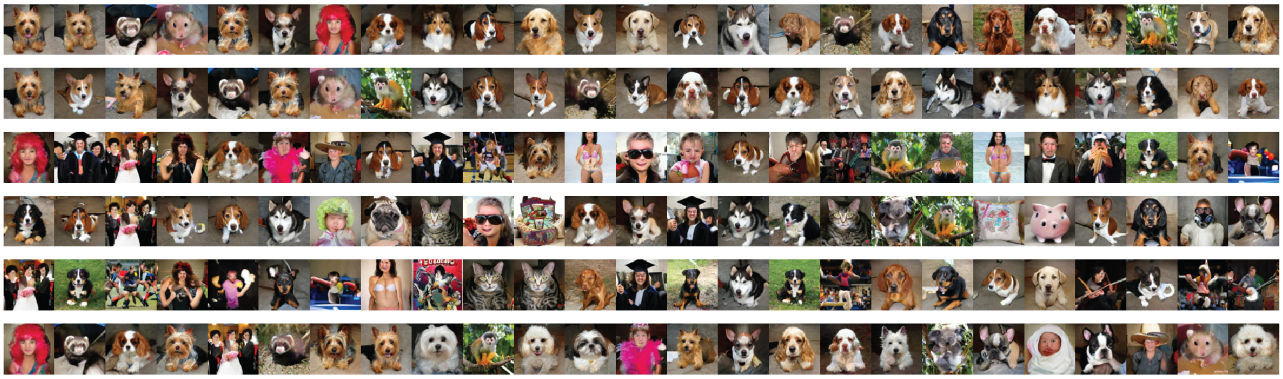

c FFA2 top 25 images

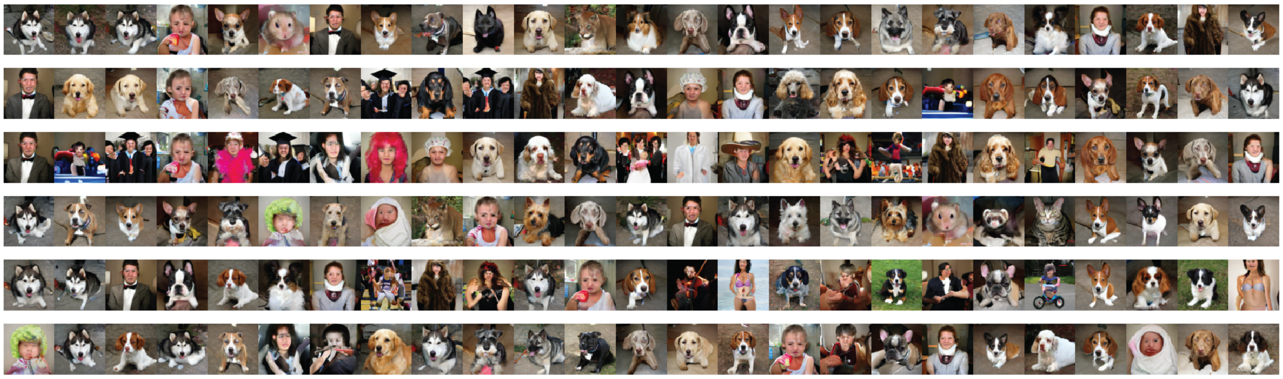

Each row represents a subject.

Supplementary Figure 7: Top 25 synthetic images using NeuroGen with linear ensemble models for NeuroGen subjects.

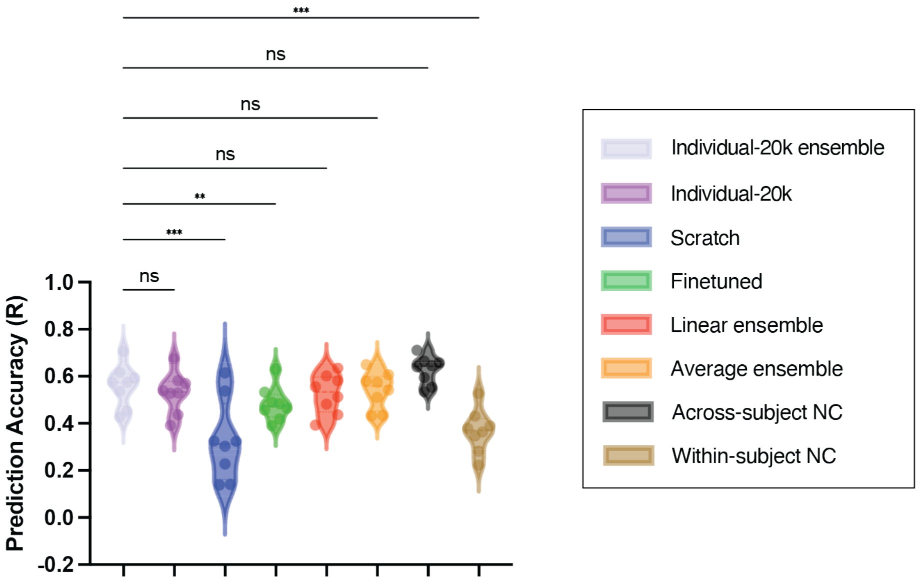

Supplementary Figure 8: Model accuracy on FFA1 comparison with an additional deep-ensembled individual-20K model (average of 7 individual-20K models trained with varied initializations) for all 8 NSD subjects.

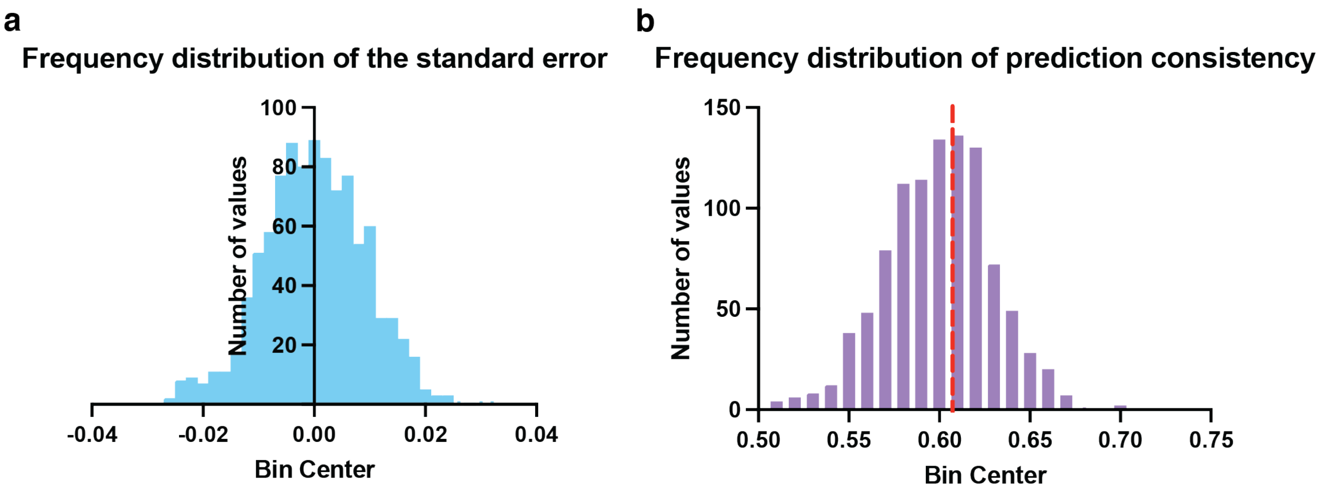

Supplementary Figure 9: Reliability of inter-subject correlation of fMRI measurement. **a** The relative error in the ISC-measurements (original value - reshuffled value)/original value, across all pairs of subjects and all regions. **b** The distribution of the individual-20K model's prediction consistency calculated using the 1000 subsamples (original value using all the data is in red).
