# Supplementary material for: Personalized visual encoding model construction with small data
Source: Commun Biol. 2022 Dec 17;5:1382. doi: 10.1038/s42003-022-04347-z (PMC9759560; doi:10.1038/s42003-022-04347-z)
Supplement: Supplementary file 3 — Description of Additional Supplementary Files [file 42003_2022_4347_MOESM3_ESM.pdf]

Description of Additional Supplementary Files

File name: Supplementary Data 1
Description: The source data behind Figure 2.

File name: Supplementary Data 2
Description: The source data behind Figure 3.

File name: Supplementary Data 3
Description: The source data behind Figure 4.

File name: Supplementary Data 4
Description: The source data behind Figure 5.
